# Atlas of Human Parasitology, 5th Edition

**DOI:** 10.3201/eid1306.070321

**Published:** 2007-06

**Authors:** Twitty J. Styles

**Affiliations:** * Union College, Schenectady, NY, USA

The 5th edition of Ash and Orihel’s Atlas of Human Parasitology is a superb, up-to-date compendium of protozoan and metazoan parasites. It also covers vectors and uncommon parasites found in humans. The authors present the material in a clear and concise manner that encourages one to delve more deeply into the structure and function of these unique and fascinating organisms. It is a must for persons interested in medical zoology and geographic medicine. Laboratory personnel, directors, and teachers who need a refresher course or additional training will find the book very valuable.

The Atlas of Human Parasitology is an essential treatise for helping to protect our citizens at home, deployed military personnel, and global travelers from parasitic infections. The quick keys to the identification of protozoans, helminths, and arthropods are helpful for distinguishing pseudoparasites from harmful ones. The labeling of various stages of the color images with letters, numbers, and arrows is extremely useful.

Attention has been given to opportunistic infections found in patients with AIDS. This book opens new vistas in helping to understand the global impact of AIDS and parasitic infections. The glossary and current references provide a ready resource for those interested in learning more about host–parasite relationships.

As an extra bonus, readers will find this edition a visual feast that integrates science and the arts. This book is highly recommended reading.

